# Rapid ethnographic appraisal of community concepts of and responses to joint pain in Kilimanjaro, Tanzania

**DOI:** 10.1136/bmjgh-2023-013245

**Published:** 2024-01-31

**Authors:** Elizabeth F Msoka, Christopher Bunn, Perry Msoka, Nateiya M Yongolo, Emma Laurie, Sally Wyke, Emma McIntosh, Blandina T Mmbaga

**Affiliations:** 1 Kilimanjaro Clinical Research Institute, Moshi, Tanzania; 2 Kilimanjaro Christian Medical University College, Moshi, Tanzania; 3 School of Social and Political Sciences, University of Glasgow, Glasgow, UK; 4 School of Geographical & Earth Sciences, University of Glasgow, Glasgow, UK; 5 School of Health & Wellbeing Social Sciences, University of Glasgow, Glasgow, UK; 6 School of Health Economics and Health Technology Assessment, University of Glasgow, Glasgow, UK

**Keywords:** Arthritis, Public Health, Qualitative study

## Abstract

**Introduction:**

Musculoskeletal disorders, experienced as joint pain, are a significant global health problem, but little is known about how joint pain is categorised and understood in Tanzania. Understanding existing conceptualisations of and responses to joint pain is important to ensure both research and interventions are equitable and avoid biomedical imposition.

**Methods:**

Rapid ethnographic appraisal was conducted in a periurban and rural community in Kilimanjaro, documenting language used to describe joint pain, ideas about causes, understandings of who experiences such pain, the impacts pain has and how people respond to it. We conducted 66 interviews with community leaders, traditional healers, community members and pharmacists.

Photographs were taken and included in fieldnotes to supplement the interview data and develop thick descriptions. Data were analysed by constant comparison using QDA Miner software.

**Results:**

Across the sample, dominant concepts of joint pain were named *ugonjwa wa baridi*, cold disease; *ugonjwa wa uzee*, old age disease; *rimatizim*, disease of the joints; and g*auti*, gout. Causes mentioned included exposure to the cold, old age, alcohol and red meat consumption, witchcraft, demons and injuries/falls. Age, gender and occupation were seen as important factors for developing joint pain. Perceived impacts of joint pain included loss of mobility, economic and family problems, developing new health conditions, death, reduction in sexual functioning and negative self-perceptions. Responses to joint pain blended biomedical treatments, herbal remedies, consultations with traditional healers and religious rituals.

**Conclusions:**

Conceptualisations of and responses to joint pain in the two communities were syncretic, mixing folk and biomedical practices. Narratives about who is affected by joint pain mirror emerging epidemiological findings, suggesting a strong ‘lay epidemiology’ in these communities. Anthropological methods can support the decolonisation of global health by decentring the imposition of English language biomedicine and pursuing synthetic, dignified languages of care.

**WHAT IS ALREADY KNOWN ON THIS TOPIC**

- African languages and associated cultural frameworks have been and are suppressed by colonial, postcolonial and global health regimes.
- No research has been done to understand how Tanzanian communities categorise and make sense of joint pain.

**WHAT THIS STUDY ADDS**

- We present the first study describing how communities in Kilimanjaro, Tanzania, make sense of and respond to joint pain.
- We present a typology of languages and concepts that circulate in two communities in Kilimanjaro about joint pain.

**HOW THIS STUDY MIGHT AFFECT RESEARCH, PRACTICE OR POLICY**

- Our methodology echoes calls for renewed attention to language in global health, following the traditions of medical anthropology, to guide research that seeks to establish equitable understandings of health problems and avoid imposing biomedical categorisation.
- The typology and our thematic analysis offer clinicians in northern Tanzania (and potentially wider Tanzania) insight into how consulting patients may talk about and understand joint pain, providing a foundation for the future development of communicative approaches that embed dignity in care.

## Introduction

Musculoskeletal Disorders (MSKD) are among the leading causes of disability worldwide.^1 2^ Those who live with MSKD face challenges such as chronic pain,^3^ increased risks of cardiometabolic disorders,^4 5^ reduced social^6^ and economic participation,^7 8^ and mental distress.^9^ Until recently, it was conventional to view MSKD, including rheumatoid arthritis specifically, as uncommon in African countries.^1 10^ This reflects reductive characterisations of African populations as ‘biologically simple’, with infectious disease positioned as the primary concern.^11^ However, epidemiological studies have established that the continent of Africa saw the most rapid increase in the incidence of MSKD between 1990 and 2017,^12^ and clinical presentations are now beginning to be characterised.^13^ Further epidemiological and clinical research is urgently needed to inform service provision, as it is clear that there is a substantial unmet burden of MSKD across the continent.

What is less clear is how diverse African communities make sense of MSK conditions, such as arthritis. Broad idioms of pain^11^ and distress^14 15^ have been documented, while work undertaken in Botswana has described how Setswana speakers talk about MSKD more specifically.^16^ These contributions aside, we do not have a cogent understanding of how painful and inflamed joints (‘joint pain’) are spoken about and imbued with meaning in different ways across the continent and its rich array of healing practices.^17^ This is likely in part due to the magnitude of the cultural and linguistic systems in which such knowledge circulates and is reproduced. It is also attributable to the impositions of biomedical approaches to disease by colonial powers, international organisations, governments, researchers and healthcare practitioners.^18^ Such impositions have silenced folk knowledge through processes of epistemic violence or ‘epistemicide’^19^ and require decolonising.^20^

The work of decolonisation in global health demands that we dismantle the structures through which the epistemic hegemony and injustices of ‘Western’ biomedicine are perpetuated.^21^ As part of an assemblage of forces, the enduring ‘coloniality’ of global health, conceived of as ‘the matrix of power relations that persistently manifests transnationally and intersubjectively despite a former colony’s achievement of nationhood’,^22^ is underpinned by biomedical impositions, mostly delivered in English. These impositions and their English language departure point not only do violence to communities but also hinder the pursuit of improved health. For example, Lwanda describes how the multisector HIV response in Malawi deployed categories with little attention to local language, causing unintended consequences which disrupted public health efforts to contain the virus.^23^ Displacing the centrality of English language biomedicine is therefore essential for decolonial efforts in global health.

To do this, researchers need to systematically engage with how people in the communities they work with make embodied experiences of illness meaningful through their everyday language and concepts.^24^ Methodological and theoretical approaches that pursue these objectives have been championed by medical anthropologists. In the discipline’s substantial corpus of work, researchers have set out how diverse African communities name and understand conditions such as diabetes,^25^ mental distress,^15^ malaria,^26^ febrile illness^27^ and HIV,^28^ critically appraising how these intersect with English language biomedicine and influence health. Underpinning these approaches is a concern with emic conceptualisations of disease—‘cultural’^29^ or ‘lay’^30^ epidemiology—through which communities make sense of and respond to bodily struggles. These emic conceptualisations form part of medical/cultural systems that draw on professional, popular and folk sectors, as Kleinman argued,^31^ or, alternatively, on traditional, modern and syncretic medical discourse, as Stoner suggests.^32^ Foregrounding everyday language (frequently silenced by colonial power in African societies)^33 34^ through which these systems are constructed and reproduced is an essential first step for avoiding biomedical imposition and the coloniality that it perpetuates.

In 1969, Tanzania was reported to have been home to 126 languages, but these languages have come under pressure from the national language of Swahili, which was established as part of the country’s postcolonial processes of modernisation and state building.^35 36^ While Swahili is now dominant in Tanzania, regional languages and dialects remain pillars of everyday life, particularly in rural communities.^37^ Consequently, any attempt to engage in meaningful and equitable dialogue with Tanzanians about painful and inflamed joints demands that the variety of sociolinguistic framings that circulate in their communities are identified and acknowledged in subsequent action. This requirement extends to those who practice medicine in Tanzania, whose sociolinguistic resources characteristically include English language biomedicine, Swahili and, at most, one other Tanzanian language.

In this paper, we describe a rapid ethnographic appraisal (REA) that aimed to characterise concepts of and responses to joint pain in the Hai district of Tanzania, as part of a wider study of MSKD prevalence and the setup of a new MSKD clinic.^38^ We aimed to provide systematic insights into these non-biomedical conceptualisations of joint pain that healthcare practitioners and researchers navigate, whether knowingly or not, as they work with community members in the Hai region who live with painful and inflamed joints. In doing so, we also aim to re-emphasise how approaches to language inspired by medical anthropology can support decolonial approaches in global health research that pursue equitable responses to health problems. To achieve these aims, we pose and respond to four research questions:

1. What are the dominant concepts of joint pain and its causes that circulate in the two communities?
2. Who do participants perceive as likely to experience joint pain?
3. How do participants perceive the impacts of joint pain on those who experience it?
4. According to participants, what types of care or treatment do people with joint pain seek?

## Methods

**Figure 1:**
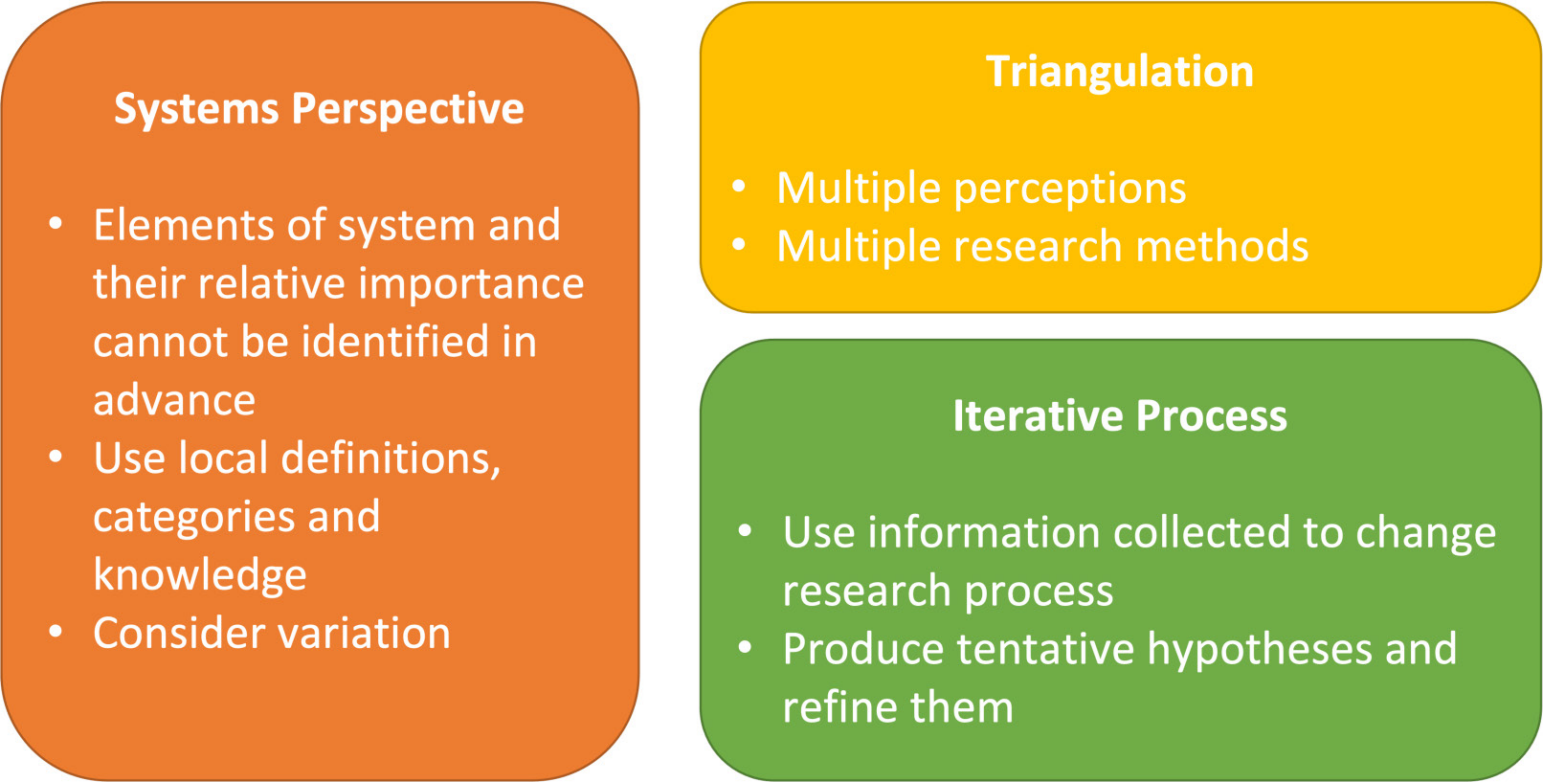
Beebe’s conceptual framework for rapid appraisal, adapted by the authors from Beebe (1995).

The study design was REA, a method well suited for intensive enquiries that aim to establish situated accounts of social understandings and practices about which researchers have little knowledge. Such designs can also facilitate more equitable connections between researchers and communities, enabling a collaborative pursuit of situated solutions to issues such as joint pain and malaria.^26 39^ The REA approach to brief ethnography blends a range of methods, commonly semistructured interviews, observations and group interviews. We deployed Beebe’s framework to guide the design of the study.^40^ The framework emphasises three core guiding concepts: employ a systems perspective, triangulate data and work iteratively (see figure 1).

The three guiding concepts are operationalised through a combination of methods and a research process that is responsive to the learning that occurs as the research develops. In our research design, we combined semistructured interviews, observation, photography and focus group discussions (FGDs), supplemented by background research via academic papers and official statistics relating to the Hai district. We deployed a constant comparison method, drawn from grounded theory, to enable iterative interpretation and systematisation of the material generated during the appraisal.^41^

### Research contexts

The study took place in two communities in the Hai district: the rural village of Lemira Kati and the periurban trading centre, Bomang’ombe. These communities were selected to provide insights into both urban and rural sociolinguistic framings relating to joint pain.

**Figure 2:**
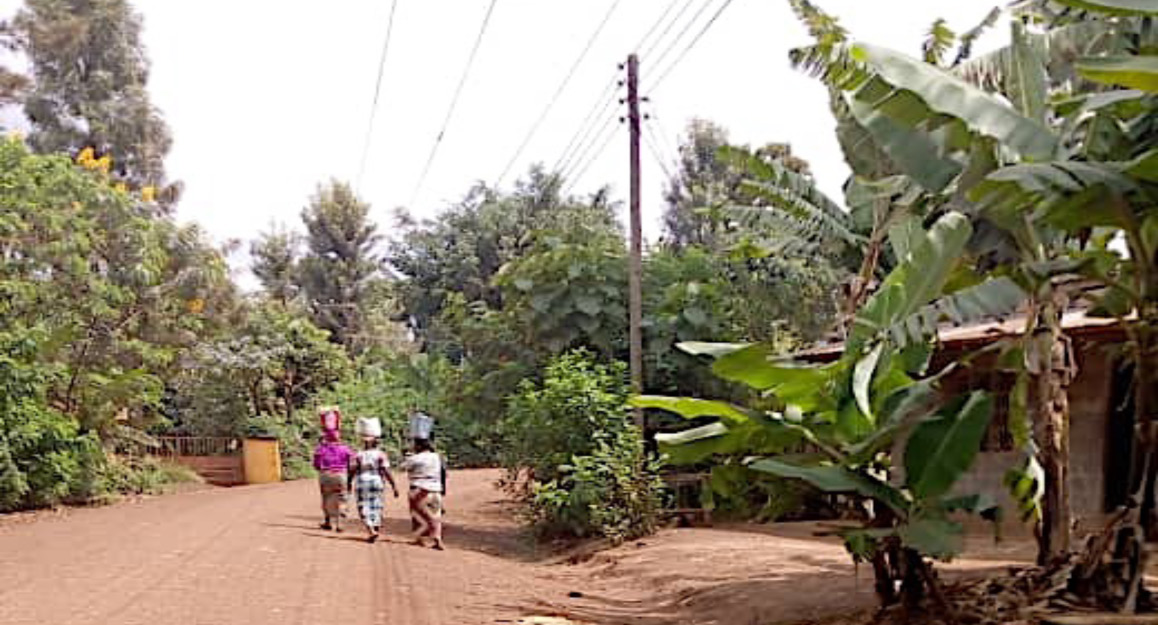
Street in Lemira Kati. Photographer: Elizabeth F Msoka.

Lemira Kati is situated on the slopes of Mount Kilimanjaro and is home to a population of approximately 1600 people, most of whom are from the Chagga tribal group.^42^ The majority of those living in Lemira Kati engage in farming, with common crops including bananas, maize, coffee and beans, which collectively produce a lush green environment, interspersed with dirt roads (see figure 2). These crops are sold along with other home-crafted produce (such as furniture) at twice-weekly markets in the town, with some farmers also travelling down the mountain to vend at larger markets. Alongside the farming community, a range of small businesses exist, including butcheries, tailors, convenience shops and motorbike taxis. Both Christians (Lutheran and Assemblies of God) and Muslims live in the village, as do those who practice folk religious beliefs.

**Figure 3:**
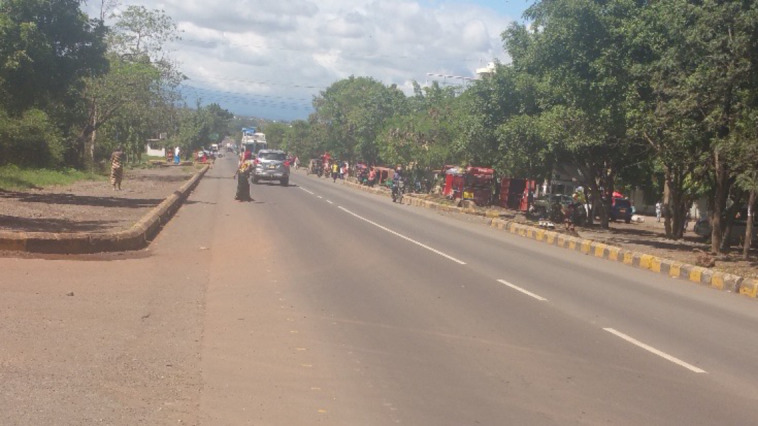
Main road in Bomang’ombe. Photographer: Elizabeth F Msoka.

By contrast, Bomang’ombe is a large, periurban trading centre, situated on the arterial road that links the cities of Moshi and Arusha and houses a diverse population of approximately 14 000 people.^42^ The bustling trading centre contains a range of retail outlets, markets, health facilities (including Hai district hospital), banks and transport hubs, as well as a petrol station, hotel, a police station and a cluster of government offices (see figure 3). The local population find employment in these facilities, engage in self-employed businesses, labour at a local sand pit where construction sand is extracted and farm nearby land. Bomang’ombe houses a variety of both Christian and Muslim traditions, as well as folk religious beliefs.

### Community entry, recruitment and sampling

REA was conducted as part of a larger cross-sectional survey of MSKD and arthritis prevalence and associated economic, quality of life and societal impacts.^38 43^ At the start of this programme of work, Tanzanian researchers (including researchers from Hai who speak Chagga and Maasai) from Kilimanjaro Clinical Research Institute led a series of community dialogue sessions with community leaders (village headmen) from across the Hai district who had participated in an established longitudinal health and demographic survey.^44^ During these sessions, the researchers set out tentative plans for the research, including REA, and sought feedback and suggestions on how the research should be implemented. These sessions took place after securing formal ethical approval from local, national and international ethics committees (see ‘Ethics Statements’ below).

Following these sessions, the REA team finalised the research design and engaged two enumerators who are employed as part of the established survey, to set up meetings with the community leaders in Lemira Kati and Bomang’ombe and who live in these communities. During these meetings, the research team explained the aims of the study and answered questions, before agreeing on procedures for conducting the study in each community.

Once community leaders had given the research team permission to conduct the study in their communities, enumerators needed to recruit potential participants. During recruitment, the enumerators and research team were guided by a theoretical sampling frame designed to elicit and engage with any sociolinguistic framings of joint pain that participants were aware of, as encouraged in REA studies.^40^ The sampling frame included targets to recruit community leaders (4), religious leaders who lead a church or mosque (8), traditional healers from one of the four broad approaches practised in Tanzania^4 45 46^ drug dispensers (4), community members 18–35 (20) and community members 35+ (20). These targets included the brief to vary by religious and tribal groupings and to be balanced equally between men and women. To operationalise these principles, enumerators constructed a sampling frame for each group we wanted to interview, identified through their established community networks. They then contacted people identified in turn in each group, sharing study information sheets and asking for consent to pass contact details to the research team.

FGD recruitment took place once the interview phase of REA was completed. We conducted three focus groups, one each for speakers of the Chagga, Pare and Maasai languages, to confirm and amend our interpretations of concepts of joint pain gathered through interviews, add new concepts and inform the language used in the cross-sectional survey that followed this study.^43^ These three language groups were selected to reflect non-Swahili groups that were expected to be most common among survey respondents. Enumerators were once again engaged to identify potential participants, providing them with study information sheets, which were also read aloud and discussed, before sharing the details of those who consented to be contacted with the research team, which then sampled to ensure that a mix of ages and genders were present in the focus groups.

### Consent and data generation

Members of the research team conducted observations and interviews during May and June 2019. FGDs were held in August and September of 2019. Before the interviews and FGDs were conducted, participants were provided with an additional copy of the study information sheet (in Swahili) and encouraged to ask questions of the researchers. Following this, all participants gave written informed consent, in the presence of a witness where requested.

Interview participants generally preferred to be interviewed at home or in their place of work, with a small number requesting they be interviewed at a neutral, private location in the community. Interviews and FGDs were conducted by EM, PM and NY and were recoded digitally, using password-protected devices. Interviews were conducted in Swahili, but participants were encouraged to describe words and phrases from other languages they speak that relate to joint pain. Interview summaries were written up in Swahili and translated to English by EM and PM, in a standardised template, using the audio recordings to ensure accuracy and to include verbatim quotations relating to focal topics. FDGs were transcribed verbatim and then translated into English by the researchers who conducted them. Both interview summaries and focus group transcripts included vernacular words and phrases relating to joint pain and inflammation that participants shared with researchers.

Observation notes inspired by a thick description approach were written for each field visit.^47^ EM and PM documented the everyday activities of those living in the two research sites, seeking to capture aspects of the context of the social actions they observed, as well as the thoughts, motivations and intentions that underpinned it (see online supplemental appendix S1 for an example).^48^ Photographs of the environment, participants and their everyday surroundings and activities were taken, with their written informed consent, to enrich what visual sociologists have called ‘the definitions of the subject’.^49^

10.1136/bmjgh-2023-013245.supp1 Supplementary data

### Data analysis

Data analysis was conducted in two phases. The first phase of analysis involved the iterative assessment of interview data during the data generation period. EM, PM and CB met on a weekly basis throughout the 8-week fieldwork period, reading interview summaries to identify vernacular terms for joint pain and inflammation, following a constant comparison method.^41^ Using an Excel spreadsheet, these terms were compiled into a typology of words and phrases participants shared with us when asked about how they speak about pain and inflammation in the joints, with separate sheets for each language they referred to. Working definitions for these words and phrases were constructed from interviewee accounts and supplemented when subsequent interviewees provided additional information. Typology entries were shared and discussed in the FGDs to check their validity with the three language groups, with contributions used to produce a final typology. We conducted a frequency analysis of the typology to establish the relative commonality of each entry. On completion of the typology, we convened a mixed group of social scientists and clinicians (NY, EM, PM, CB, CK and RW) to review entries for the likelihood that they related to joint pain. Results from this review were passed to survey designers to inform prompts for use among respondents participating in a cross-sectional survey to estimate the prevalence of MSKD in the Hai district.^38^

The second phase of analysis took the form of a reflexive thematic analysis of the interview notes, thick descriptions and photographs.^50^ EM, PM and CB independently and inductively coded six interviews, thick descriptions and accompanying photographs before comparing approaches and collaboratively constructing a preliminary coding framework which was used to code the full dataset and supplemented with new codes as necessary. Following this, EM, PM and CB read and re-read coding reports and transcripts to generate and develop themes that responded to the overall research objective to characterise understandings of and responses to joint pain in the two research sites. These themes were organised to address the four specific research questions. For research question one, the thematic analysis was triangulated^51^ with the frequency analysis conducted on typology entries to establish dominant concepts of joint pain. Coding was done using QDA Miner (V.5.0). This software was chosen due to the availability of a free-to-use ‘Lite’ version, ensuring that all involved in the research were able to develop transferable skills that were not restricted to an expensive subscription-only tool.

### Patient and public involvement

At the start of the National Institute of Health Research (NIHR)-funded study in which this enquiry was nested, stakeholder consultations with community leaders, community members, healthcare professionals and representatives from the Ministry of Health were held in Kilimanjaro. These events focused on raising awareness of the research and on gathering feedback on the design and implementation of data collection procedures, including those we report on in this paper. During these events, community members contributed to defining the procedures that enumerators should follow when making contact in relation to the present study, as well as procedures used in the wider study. We were unable to identify a patient group for people living with arthritis/MSKD in Tanzania, and to the best of our knowledge, none exists. An author reflexivity statement is provided in online supplemental appendix S2.

10.1136/bmjgh-2023-013245.supp2 Supplementary data

## Findings

The research team conducted 66 interviews over the course of 2 months, successfully recruiting participants for all the social groups represented in the sampling frame (see online supplemental appendix S3). Among the traditional healers we rectruited, all four participants’ practices fit the category of ‘herbalists’.^45 46^ The team wrote 24 observation notes and took 169 photographs. In what follows, we present findings under headings that relate to our four research questions. Findings were common across the two communities, except where noted.

10.1136/bmjgh-2023-013245.supp3 Supplementary data

### What are the dominant concepts of joint pain and its causes?

Our rapid appraisal generated a typology that contained 151 words/phrases which participants associated with joint pain, spread across 10 languages (see online supplemental appendix S4). Two languages were identified as dominant: Swahili (63 words/phrases) and Chagga (56). The eight other languages included Iraq (7), Maasai (6), Sambaa (5), Gogo (4), Kwere (3), English (3), Pare (2) and Rangi (2). A mixed team of social scientists and clinicians reviewed the typology and judged 101 entries to be likely related to joint pain and 50 unlikely to be related to joint pain. The 101 entries considered likely to be related to joint pain were passed to the survey design team and used to develop survey prompts for use with participants who do not speak Swahili.

10.1136/bmjgh-2023-013245.supp4 Supplementary data

Through a triangulation of our thematic and frequency analyses, we established four dominant conceptions of joint pain circulating in the two communities: a *cold disease*, an *old age disease*, rheumatism and gout. The connection between joint pain and coldness was particularly strong and was referred to in 18 entries in the typology. In Swahili, participants described a condition they referred to as *ugonjwa wa baridi* (‘cold disease’) and also *baridi yabisi* (cold and inflexible/hard/stiff). Among Chagga speakers, *ndwari ya mbeho*, *ndori ambio* and *ugonjwa wa mbeo* (variations of ‘cold disease’) were described as directly referring to joint pain. A range of other concepts were shared by participants which indirectly related joint pain to coldness for example, in Maasai, the word *embolosi* was reported to refer to waist pain that can be brought on by cold conditions.

Our analysis brought out multiple dimensions of the perceived relationship between ‘coldness’ and ‘cold disease’. Exposure to the cold was a common explanation, with participants describing how walking barefoot or working in cold and damp conditions could lead to cold disease: ‘…joints are frozen, the disease is due to working on the water’ (religious leader, urban, M51). The link between coldness and cold disease was also described as a seasonal one, with cold disease said to occur ‘…during this time of rain and cold season’ (community member, rural, M61). Finally, coldness was also positioned as a quality of an ageing body:

People from 50 years of age and above… at this age the body’s energy is declining, so the body starts to become cold which leads to pains, especially in the joints. Religious leader, rural, F37

As this account reveals, older people are thought to be particularly vulnerable to joint pain due to their ‘declining’ bodies which lead to the ‘cooling’ of the body and, therefore, cold disease. Low levels of activity were also perceived by some to have the potential to cause joint pain during middle age, particularly among those whose routines required them ‘settling in one place’ (community member, urban, M27).

Such accounts, combined with frequency analysis of our typology, led us to thematically characterise joint pain as perceived to be an *old age disease*. Specifically, eight participants referred to joint pain, using Swahili, as *ugonjwa wa uzee* (disease of old age). While no other sociolinguistic grouping characterised joint pain as *old age disease* in such direct terms, references to joint pain as a condition of old age were recurrent in how people explained a range of concepts. For example, in Chagga, the phrase *maoko alemakaside* (severe hand pain) was positioned as a phrase commonly used by older people; in Iraq, the phrase *dandatili* (back pain) was also attributed to older people; and in Maasai, the aforementioned concept of *embolosi* was also related to old age.

Alongside the conceptions of joint pain as a *cold disease* and an *old age disease*, concepts related to English language medical terms were also referred to, representing an intersection of ‘professional’ and ‘popular’ sectors of local healthcare.^31^ Rheumatism, often pronounced as *rimatizim*, was referred to in six interviews and characterised as ‘a disease which affect the bones’ (community member, rural, F51), which was sometimes linked to ‘cold disease’. Another medical term, gout (or *gauti*), was referred to by five interviewees, who characterised the condition by describing symptoms of pain affecting the lower limbs and knees. Accounts of *gauti* were more common in the urban than rural areas and were perceived to be caused by eating goat meat and drinking beer, as well as ‘dry’ joints.

Accompanying these dominant conceptions of joint pain, and their associated causes, were a range of causes which were linked to both the dominant concepts we have described and concepts mentioned less frequently. First, joint pain was often connected to past injuries to the bones. Second, six participants spoke of *uchawi* (witchcraft) as a cause of joint pain, as a consequence of damaging another’s interests (eg, stepping on an egg belonging to another person) or as a direct attack from someone who wishes you ill. Finally, some positioned joint pain as the consequence of demonic possession or sexual transgressions.

### Who gets joint pain?

Our analysis produced three themes that capture how participants made sense of who in their community is likely to suffer from joint pain. First, as set out above in our description of *ugonjwa wa uzee*, joint pain was frequently positioned as *an old age-related condition*.

Alongside older people, participants also characterised joint pain as *a women’s disease*. The accounts which elaborated on this perception tended to relate this to gendered divisions of labour:

Women are the ones getting joint pain more than men, since they are doing hard work, in many activities. Even if it’s raining you will find them still working and they complain about pain in their legs and hands because of cold. Community member, rural, F22

The household, family and farming work that predominantly falls to women in these two communities was seen to make them more susceptible to joint pains caused by the entry of coldness into their bodies. While such perceptions dominated, a few male participants sought to make sense of men’s joint pain and, in doing so, suggested that men were more likely to experience such pain due to the farming work they undertook.

The final theme generated through our analysis captured an account of how *occupations can lead to joint pain*. As accounts of the gendered nature of joint pain began to illustrate, participants made strong links between the types of work that people do and their likelihood of suffering from joint pain. Some gave the example of ‘butchermen’ as an occupation that is likely to experience joint pain, because butchers work in the cold and chop meat, causing pains in the wrist. Others suggested that those who move up and down Mount Kilimanjaro as part of their work, either as ‘porters’ for climbers or transporting goods and produce on foot, were likely to experience joint pain. In both examples, exposure to the cold was emphasised.

### How does joint pain affect those who experience it?

Accounts of the impacts of joint pain on the lives of those whom it afflicts were rich and varied. Our analysis generated three themes and noted a range of ‘other’ accounts which we will also summarise briefly. Central to how community members made sense of the effects, joint pain was *the loss of mobility*.

People with joint pain cannot move fast, cannot work… it is also difficult for a patient to sit when they want to use the toilet, which may affect them and make them feel bad. Community member, urban, M24

Participants’ accounts commonly described how joint pain limited movement, inhibiting the most basic and essential activities, such as using the toilet and moving to do work.

The consequences of limited mobility were often linked in participants’ accounts to *economic and family suffering*.

People who suffer from joint pain cannot do anything in the family. For example, if he was a breadwinner, the family suffer and they will also need to take care of him. If the patient is a mother, they will need to hire a person who can do what she used to do, so to many families it becomes extra work or a burden. Community member, rural, M41

In accounts such as this, participants set out how joint pain and the limitations it introduced in the bodies of those who live with it produce a cascade of problems for families, including lost income and increased household and caring work, producing a ‘burden’.

A third theme created through our analysis captures how participants viewed joint pain as *a cause of new conditions*.

Joint pain can cause development of other conditions, especially for older people – when they have back pain, they will experience fever. Ccommunity member, urban, F53

A person [living with joint pain] may develop other diseases such as blood pressure. Religious leader, rural, M66

In such accounts, then, joint pain is seen as driving new health problems, particularly in old age.

During our analysis, we also noted that some participants viewed joint pain as closely linked to death, although the perceived causality of this link remained opaque in the data. Some participants also spoke about joint pain as leading to reduced sexual desire and prowess, while others spoke about joint pain leading to negative self-perceptions, as alluded to above, when the participants noted that those who cannot go to the toilet due to their joint pain would ‘feel bad’.

### What do people do about joint pain?

Community members in Lemira Kati and Bomang’ombe spoke about a range of responses to joint pain, which we captured using three themes. The first of these described perceptions of how those living with joint pain *use medical treatments*.

People with joint pain attend hospital to see the medical doctors for the treatment and they usually get Indocid [Indomethacin] and Brufen [ibuprofen]. Community leader, urban, F65

The use of non-steroidal anti-inflammatory drugs (NSAIDs) to treat joint pain was referred to frequently in the accounts generated during the rapid appraisal, but access to medical doctors was often viewed as costly. For example, one community member described how they access medication without consultation:

I buy the medication from the pharmacies because I don’t have the Health Insurance Card and I cannot always afford to pay the hospital. Community member, urban, F40

The use of pharmaceuticals without consultation was also described by drug dispensers:

When patients come to us to buy medicine we do advise them to attend hospital, because not all the joint pains are due to cold. Drug dispenser, rural, F42

**Figure 4:**
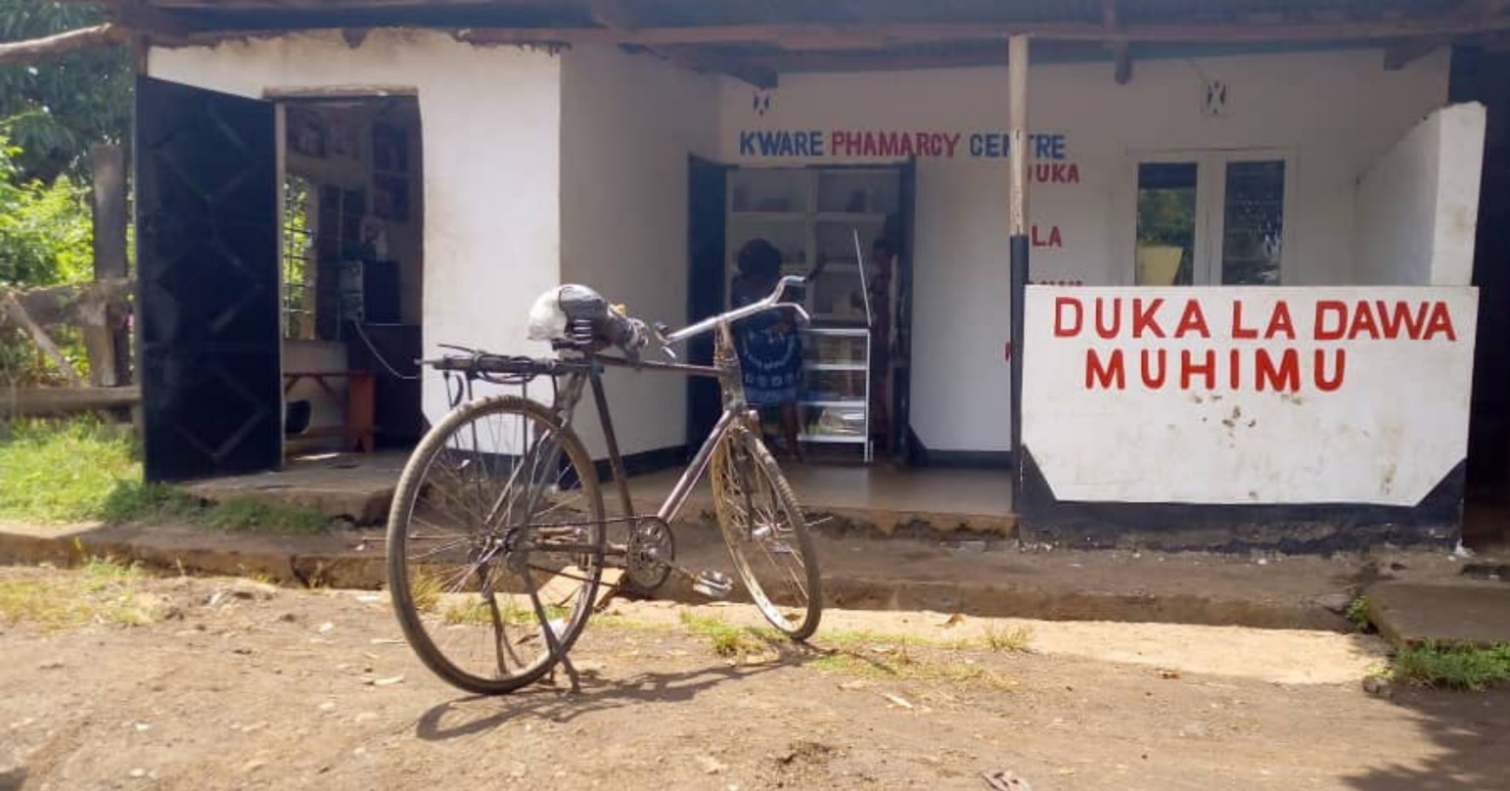
Drug dispensary in Lemira Kati. Photographer: Elizabeth F Msoka.

Our analysis suggests that medical responses to joint pain are common, with NSAIDs widely used, but that these drugs are commonly used without medical supervision, purchased directly from dispensaries (eg, figure 4).

Alongside medical treatments, participants described the use of multiple approaches to healing. Our thematic analysis highlighted the common perception that those living with joint pain *use herbal remedies*. This was emphasised in accounts from community leaders and members, but traditional healers offered particular insight into the use of herbal medicines:

*Kifuku –* these are roots which are boiled and drunk from a cup three times a day… they are used to treat joint pain…

Most people seek traditional medicine. However, some people do attend hospital and they get cold disease medicine which makes the bones soft… if that happens a small accident can break the leg Traditional healer, rural, M59

In these accounts, traditional healers position their solutions with confidence. *Kifuku* roots are asserted as a way to ‘treat’ joint pain, and such ‘traditional medicine’ is contrasted to biomedical approaches to treating ‘cold disease’, which is said to thin the bones. The latter point is particularly interesting, given its potential reference to corticosteroid treatments often used to treat arthritis, which can indeed thin the bones and increase the risk of fracture.

Alongside medical and herbal treatments, participant accounts described how *metaphysical solutions* are commonly sought for joint pain. These metaphysical approaches took two forms. First:

In the community people believe that there is witchcraft. Many people with chronic pain or disease attend the witchdoctor to find out why they haven’t been cured. Community member, rural, M31

As well as seeking solutions from ‘witch doctors’, interviewees also described how some turn to prayer:

Rimatizim is a disease of possession by demons… hence they treat it with prayers. Community member, rural, F50

Following conceptions of joint pain that position it as a witchcraft or demonic phenomenon, then community members described solutions originating from within the sociocultural systems that address these metaphysical domains.

Our analysis suggested that these three discrete responses to joint pain are, in fact, often pursued in combination, either simultaneously or sequentially. This syncretic approach to treating joint pain is driven by a range of factors. Participants commonly described hospital treatment as expensive, traditional medicine and basic painkillers as more affordable and metaphysical solutions as essential, given the role god and/or spiritual forces play in shaping health.

## Discussion

REA set out to characterise how two communities in the Hai district of Tanzania make sense of, and respond to, joint pain. We found a diverse, rich sociolinguistic landscape which predominantly characterises joint pain as a *cold disease* and an *old age disease*. Coldness was positioned as an environmental, seasonal and age-related property, which drives joint pain. The centrality of ‘coldness’ and seasonality in concepts and explanations of joint pain echoes conceptualisations of malaria in other African countries.^52 53^ The importance of ‘coldness’ can also be understood in relation to ‘hot-cold’ theories of illness that suggest illnesses are intimately connected to bodily exposure to hot and cold elements, which anthropologists have identified across a range of cultures.^26 54 55^ This wider frame of reference suggests that joint pain is likely to be one of a number of *cold diseases* and that this category is not exclusive to joint pain.^26^ While this may appear to limit the utility of the concept for informing clinical practice and encounters, clinician engagement with ‘hot-cold’ systems can support holistic care for patients, by acknowledging and working with the practices they engender.^56^

REA found that participants positioned women and older people as most likely to suffer from joint pain. This finding suggests that the two communities have a clear ‘lay epidemiology’, to borrow a concept from medical sociology,^30^ as these observations map onto both situated and global epidemiology, which position women and the elderly as more at risk of MSKD.^57 58^ This ‘lay’ or ‘cultural’ epidemiology also intersects with ‘hot-cold’ approaches to disease, notably with the elderly positioned as living in ‘cooling’ bodies.^59^ This intersection extended to usages of the English language medical terms rheumatism and gout, with the former related to coldness and the latter to consumption of (hot) red meat and alcohol.

The circulation of these terms ‘rheumatism’ and ‘gout’ also reveals that the well-established approaches of Kleinman^31^ and Stoner^32^ can usefully inform our understanding of how participants make sense of joint pain. As it is clear that while these terms may originate from the ‘professional sector’ or ‘modern’ medicine, they have been integrated into ‘popular’ discourse, fused with ‘folk’ theories and remade as ‘syncretic’. These syncretic characterisations of joint pain also manifested in the responses to joint pain described by our participants. Participants described those within their community living with joint pain responding to these conditions using a syncretic mix of biomedical, herbal and metaphysical treatments. Yet this mixed approach cannot be reduced to syncretic conceptualisation, as participants described the cost of biomedical treatments as a factor in treatment choice, which included the common practice of self-medicating or consulting at pharmacies.^60^ The influence of cost on treatment, as well as rude interactions with healthcare professionals, is widely reported in studies of chronic illness across African countries.^61^

The anthropologically inspired typology method we used as part of this REA enabled us to reconstruct multidimensional community conceptions of, and responses to, joint pain. By centring the sociolinguistic traditions often silenced in global health research,^22^ we were able to dialogue with participants about joint pain without engaging in medical imposition, ensuring we respected their right to ‘name the world’.^62^ In this way, the typology method we adopted, built on the traditions of medical anthropology,^27^ echoes recent reminders of the importance of centring language in global health research,^63 64^ especially as part of a wider commitment to decolonising research practice and avoiding epistemic injustice,^21^ and of working pluriversally.^65^ This is particularly relevant across the African continent, where European languages have displaced and disenfranchised local languages, processes which global health researchers and practitioners have perpetuated.

Through our REA, we were able to identify the range of impacts that community members commonly perceived joint pain to have. These included loss of mobility, economic and family disruption and the cause of new conditions. Such findings align with literature from across the globe that documents the wide-ranging consequences joint pain and MSKD have in the lives of those who live with it.^66^ While our study does not offer immediate suggestions to address these impacts, it does provide a foundation for future work that emphasises what others have called ‘communicative care’.^63^ Specifically, it offers the clinicians and researchers who serve these communities insight into how joint pain is made sense of and responded to, providing the opportunity to create ‘synthetic’ languages of care that support dignity and overcome the coloniality of biomedicine.^64^ A starting point for this endeavour could be to systematically study how the ‘hot-cold’ approach to joint pain that we have described can be harnessed to support clinical encounters and the wider social relations in which such encounters are situated.^56 67^ Our study also identifies the range of healers who are consulted beyond medical professionals, including pharmacists, traditional healers and religious leaders. Given the existing roles played by these groups, future provision for people living with joint pain in the region should be developed in collaboration with them, requiring bidirectional trust grounded in respect for diverse framings of joint pain.^68^

### Strengths and limitations

Our study has a range of strengths. We spoke with a sizeable and diverse group of participants, varied by social standing, location, gender, occupation and age, providing our research with a robust sample through which to develop our analysis. Our analysis was rigorous and grounded in iterative and routine reflection on the accounts offered by our participants. The findings of the study were also used to support the design of a subsequent community survey and the establishment of a new clinic for people with MSKD, ensuring that it was culturally and linguistically optimal.

However, we acknowledge that our work was also limited by a number of factors. Conducting data collection in Swahili to elicit language from nine language groups likely influenced the range and depth of terms and phrases we were able to document. The study would undoubtedly have benefitted from including researchers from all nine linguistic traditions, instead of being limited to the two dominant traditions, Swahili and Chagga. The translation processes involved in the research are also likely to have limited our ability to understand nuance in seven language groups, with the use of Swahili and English language as mediums for capture and analysis contributing to this loss of nuance. Additionally, the rapid nature of REA research does not enable the same depth of understanding as traditional, longer-term, ethnographic research. Relatedly, our use of principles drawn from thick description was constrained by the time available to write each note, resulting in notes that did not fully capture emotions and rich meanings.^48^

## Conclusion

This study has documented the range of understandings of and responses to joint pain that circulate in two communities in the Hai district of Tanzania. Understandings and responses were characterised by a syncretic approach, grounded in ‘hot-cold’ approaches to illness, mapped closely onto existing epidemiological accounts and suggest that joint pain brings significant burden to those who live with it, as well as their social networks. Anthropological approaches that foreground diverse languages enable us to avoid biomedical impositions and can contribute to the decolonisation of global health research and practice by decentring the imposition of English language biomedicine. Such approaches also provide a foundation for synthetic, dignified languages of care that are holistic and engage with the systems through which patients and their social relations make sense of their illness.

## Data Availability

Data are available upon reasonable request. Data are available upon reasonable request to the corresponding author.

## References

[1] Briggs AM, Cross MJ, Hoy DG, et al. Musculoskeletal health conditions represent a global threat to healthy aging: a report for the 2015 World Health Organization World Report on ageing and health. Gerontologist 2016;56 Suppl 2:S243–55. 10.1093/geront/gnw002 26994264

[2] Murray CJL, Barber RM, Foreman KJ, et al. Global, regional, and national disability-adjusted life years (DALYs) for 306 diseases and injuries and healthy life expectancy (HALE) for 188 countries, 1990–2013: quantifying the epidemiological transition. Lancet 2015;386:2145–91. 10.1016/S0140-6736(15)61340-X 26321261 PMC4673910

[3] Cimmino MA, Ferrone C, Cutolo M. Epidemiology of chronic musculoskeletal pain. Best Pract Res Clin Rheumatol 2011;25:173–83. 10.1016/j.berh.2010.01.012 22094194

[4] Zhang K, Jia Y, Wang R, et al. Rheumatoid arthritis and the risk of major cardiometabolic diseases: a mendelian randomization study. Scand J Rheumatol 2023;52:335–41. 10.1080/03009742.2022.2070988 35658786

[5] Jafri K, Bartels CM, Shin D, et al. Incidence and management of cardiovascular risk factors in psoriatic arthritis and rheumatoid arthritis: a population‐based study. Arthritis Care Res 2017;69:51–7. 10.1002/acr.23094 PMC5191972 27696731

[6] Bay LT, Ellingsen T, Giraldi A, et al. “To be lonely in your own loneliness”: The interplay between self-perceived loneliness and rheumatoid arthritis in everyday life: a qualitative study. Musculoskeletal Care 2020;18:450–8. 10.1002/msc.1480 32491275

[7] Schofield DJ, Callander EJ, Shrestha RN, et al. How co-morbidities magnify the effect of arthritis on labour force participation and economic status: a costs of illness study in Australia. Rheumatol Int 2014;34:481–9. 10.1007/s00296-014-2967-5 24562914

[8] Hoy D, Toole MJ, Morgan D, et al. Low back pain in rural Tibet. Lancet 2003;361:225–6. 10.1016/S0140-6736(03)12254-4 12547548

[9] Fakra E, Marotte H. Rheumatoid arthritis and depression. Jt Bone Spine 2021;88:105200. 10.1016/j.jbspin.2021.105200 33932572

[10] Hodkinson B, Tikly M, Adebajo A. Rheumatoid arthritis in the developing world: stepping up to the challenge. Clin Rheumatol 2014;33:1195–6. 10.1007/s10067-014-2690-3 24894106

[11] Livingston J. Improvising medicine: an African oncology ward in an emerging cancer epidemic. Duke University Press, 2012. 10.1080/13648470.2014.929634 24962205

[12] Jin Z, Wang D, Zhang H, et al. Incidence trend of five common musculoskeletal disorders from 1990 to 2017 at the global, regional and national level: results from the global burden of disease study 2017. Ann Rheum Dis 2020;79:1014–22. 10.1136/annrheumdis-2020-217050 32414807

[13] Kuo C, Black L, Kelly C. E036 Arthritis in East Africa: an observational study. Rheumatology 2023;62:3–9. 10.1093/rheumatology/kead104.285

[14] Mendenhall E, Kim AW. Rethinking idioms of distress and resilience in anthropology and global mental health, in global mental health ethics. In: Dyer AR, Kohrt BA, Candilis PJ Editors. Cham: Springer International Publishing, 2021: 157–70.

[15] Mendenhall E, Rinehart R, Musyimi C, et al. An ethnopsychology of idioms of distress in urban Kenya. Transcult Psychiatry 2019;56:620–42. 10.1177/1363461518824431 30672722

[16] Hondras M, Myburgh C, Hartvigsen J, et al. Botlhoko, botlhoko! how people talk about their musculoskeletal complaints in rural Botswana: a focused ethnography. Glob Health Action 2015;8:29010. 10.3402/gha.v8.29010 26689457 PMC4685300

[17] Mokgobi MG. Understanding traditional African healing. Afr J Phys Health Educ Recreat Dance 2014;20:24–34. 26594664 PMC4651463

[18] Tilley H. Medicine, empires, and ethics in Colonial Africa. AMA J Ethics 2016;18:743–53. 10.1001/journalofethics.2016.18.7.mhst1-1607 27437825

[19] Santos B de S. Epistemologies of the South: Justice against epistemicide. 2015: Routledge,

[20] Büyüm AM, Kenney C, Koris A, et al. Decolonising global health: if not now, when? BMJ Glob Health 2020;5:e003394. 10.1136/bmjgh-2020-003394 PMC7409954 32759186

[21] Bhakuni H, Abimbola S. Epistemic injustice in academic global health. Lancet Glob Health 2021;9:e1465–70. 10.1016/S2214-109X(21)00301-6 34384536

[22] Richardson ET. On the coloniality of global public heath. Med Anthropol Theory 2019;6:101–18. 10.17157/mat.6.4.761 37588113 PMC10430880

[23] Lwanda JL. Politics, culture, and medicine in Malawi. 2004: Kachere Series,

[24] Konadu K. Medicine and anthropology in twentieth century Africa: akan medicine and encounters with (medical) anthropology. Afr Stud Q 2008:10.

[25] Awah PK, Unwin NC, Phillimore PR. Diabetes mellitus: Indigenous naming, indigenous diagnosis and self-management in an African setting: the example from Cameroon. BMC Endocr Disord 2009;9:5. 10.1186/1472-6823-9-5 19224650 PMC2661081

[26] Sissoko B, Rafiq MY, Wang JR, et al. Social representations of malaria in a southern malian community: an ethnographic qualitative study. Malar J 2022;21:276. 10.1186/s12936-022-04298-0 36175914 PMC9523929

[27] Winch PJ, Makemba AM, Kamazima SR, et al. Local terminology for febrile illnesses in Bagamoyo district, Tanzania and its impact on the design of a community-based malaria control programme. Soc Sci Med 1996;42:1057–67. 10.1016/0277-9536(95)00293-6 8730911

[28] Norton B, Mutonyi H. Languaging for life: African youth talk back to HIV/AIDS research. Lang Policy 2010;9:45–63. 10.1007/s10993-009-9150-y

[29] Weiss MG. Cultural epidemiology: an introduction and overview. Anthropol Med 2001;8:5–29. 10.1080/13648470120070980

[30] Davison C, Smith GD, Frankel S. Lay epidemiology and the prevention paradox: the implications of coronary candidacy for health education. Sociol Health Illn 1991;13:1–19. 10.1111/j.1467-9566.1991.tb00085.x

[31] Kleinman A. Concepts and a model for the comparison of medical systems as cultural systems. Soc Sci Med 1978;12:85–93. 10.1016/0160-7987(78)90014-5 358402

[32] Stoner BP. Understanding medical systems: traditional, modern, and syncretic health care alternatives in medically pluralistic societies. Med Anthropol Q 1986;17:44–8. 10.1111/j.1937-6219.1986.tb01021.x

[33] Mignolo W. Local histories/global designs: coloniality, subaltern knowledges, and border thinking. Princeton University Press, 2012.

[34] Fanon F. Black skin, white masks. Grove press, 2008.

[35] Legère K. Language shift in Tanzania. Contributions to the Sociology of Language 1992;64:99. 10.1515/9783110870602.99

[36] Batibo H. The fate of ethnic languages in Tanzania. Contributions to the Sociology of Language 1992;64:85. 10.1515/9783110870602

[37] Myhre KC. Returning life: language, life force and history in kilimanjaro. Vol. 32. Berghahn Books, 2017.

[38] Mmbaga BT et al. Musculoskeletal (MSK) disorders with arthritis screening in Tanzania: new insights into the growing clinical, economic and societal burden of non-communicable disease. Tanzania Public Health Bulletin 2023;10:8–14.

[39] Sangaramoorthy T, Kroeger KA. Rapid ethnographic assessments: a practical approach and toolkit for collaborative community research. Routledge, 2020.

[40] Beebe J. Basic concepts and techniques of rapid appraisal. Hum Organ 1995;54:42–51. 10.17730/humo.54.1.k84tv883mr2756l3

[41] Hallberg L-M. The “core category” of grounded theory: making constant comparisons. Int J Qual Stud Health Well-being 2006;1:141–8. 10.1080/17482620600858399

[42] Statistics NBo. Census data: village statistics. Dodoma: National Bureau of Statistics, 2012.

[43] Kilonzo KG. Estimating the prevalence, quality of life and societal impacts of arthritis in Tanzania: protocol for a mixed methods study. Protocols.io, 2023.

[44] Kitange HM, Machibya H, Black J, et al. Outlook for survivors of childhood in sub-Saharan Africa: adult mortality in Tanzania. Adult morbidity and mortality project. BMJ 1996;312:216–20. 10.1136/bmj.312.7025.216 8563587 PMC2349992

[45] Mshiu EN, Chhabra SC. Traditional healers and health care delivery in Tanzania. Trop Doct 1982;12:142–3. 10.1177/004947558201200319 7112680

[46] Gessler MC, Msuya DE, Nkunya MH, et al. Traditional healers in Tanzania: sociocultural profile and three short portraits. J Ethnopharmacol 1995;48:145–60. 10.1016/0378-8741(95)01295-o 8719975

[47] Geertz C. The interpretation of cultures. Vol. 5043. Basic books, 1973.

[48] Ponterotto JG. Brief note on the origins, evolution, and meaning of the qualitative research concept thick description. Qual Rep 2006;11:538–49. 10.46743/2160-3715/2006.1666

[49] Harper D. Visual sociology: expanding sociological vision. Am Soc 1988;19:54–70. 10.1007/BF02692374

[50] Braun V, Clarke V. Reflecting on reflexive thematic analysis. Qual Res Sport Exerc Health 2019;11:589–97. 10.1080/2159676X.2019.1628806

[51] Denzin NK. Triangulation. The blackwell encyclopedia of sociology. 2007.

[52] Deressa W, Ali A, Enquoselassie F. Knowledge, attitude and practice about malaria, the mosquito and antimalarial drugs in a rural community. Ethiop J Health Dev 2003;17:99–104. 10.4314/ejhd.v17i2.9849

[53] Dugas M, Dubé E, Bibeau G. Translating malaria as sumaya: Justified convention or inappropriateness? Anthropol Med 2009;16:307–18. 10.1080/13648470903183487 27269912

[54] Brown PJ. Part III: cultural adaptations to endemic malaria in Sardinia. Med Anthropol 1981;5:313–39. 10.1080/01459740.1981.9986991

[55] Weller S. New data on intracultural variability: the hot-cold concept of medicine and illness. Hum Organ 1983;42:249–57. 10.17730/humo.42.3.v485x5npq050g748

[56] Harwood A. The hot-cold theory of disease. Implications for treatment of Puerto Rican patients. JAMA 1971;216:1153–8. 4325136

[57] Safiri S, Kolahi AA, Hoy D, et al. Global, regional and national burden of rheumatoid arthritis 1990-2017: a systematic analysis of the global burden of disease study 2017. Ann Rheum Dis 2019;78:1463–71. 10.1136/annrheumdis-2019-215920 31511227

[58] Yongolo NM, Halliday J, Bunn C, et al. Estimating prevalence and predictors of musculoskeletal disorders in tanzania – A pilot study[Preprint]. Res Sq 2022. 10.21203/rs.3.rs-1645605/v1 PMC10998254 38586069

[59] Cohen L. Toward an anthropology of senility: anger, weakness, and Alzheimer’s in Banaras, India. Med Anthropol Q 1995;9:314–34. 10.1525/maq.1995.9.3.02a00030 8542437

[60] Chipwaza B, Mugasa JP, Mayumana I, et al. Self-medication with anti-malarials is a common practice in rural communities of Kilosa district in Tanzania despite the reported decline of malaria. Malar J 2014;13:1–11. 10.1186/1475-2875-13-252 24992941 PMC4087197

[61] Zimmermann M, Bunn C, Namadingo H, et al. Experiences of type 2 diabetes in sub-Saharan Africa: a scoping review. Glob Health Res Policy 2018;3:25. 10.1186/s41256-018-0082-y 30214942 PMC6134599

[62] Freire P. Pedagogy of the oppressed (revised), Vol 356. New York: Continuum, 1996: 357–8.

[63] Arnold L, Black SP. How communicative approaches enrich the study of care. Taylor & Francis, 2020: 573–81. 10.1080/01459740.2020.1814285 33035105

[64] Rafiq MY, Krugman DW, Bapumia F, et al. Kansa talk: mapping cancer terminologies in Bagamoyo, Tanzania towards dignity-based practice. BMJ Glob Health 2023;8:e012349. 10.1136/bmjgh-2023-012349 PMC10432665 37580100

[65] Affun-Adegbulu C, Adegbulu O. Decolonising global (Public) health: from western universalism to global pluriversalities. BMJ Glob Health 2020;5:e002947. 10.1136/bmjgh-2020-002947 PMC7443258 32819916

[66] Daker-White G, Donovan J, Campbell R. Redefined by illness: meta-ethnography of qualitative studies on the experience of rheumatoid arthritis. Disabil Rehabil 2014;36:1061–71. 10.3109/09638288.2013.829531 24001261

[67] Arnold L. Cross-border communication and the enregisterment of collective frameworks for Care. Med Anthropol 2020;39:624–37. 10.1080/01459740.2020.1717490 32049550

[68] Krah E, de Kruijf J, Ragno L. Integrating traditional healers into the health care system: challenges and opportunities in rural Northern Ghana. J Community Health 2018;43:157–63. 10.1007/s10900-017-0398-4 28681282 PMC5767209

