# Supplementary material for: Rapid ethnographic appraisal of community concepts of and responses to joint pain in Kilimanjaro, Tanzania
Source: BMJ Glob Health. 2024 Jan 31;9(1):e013245. doi: 10.1136/bmjgh-2023-013245 (PMC10831465; doi:10.1136/bmjgh-2023-013245)
Supplement: Supplementary data [file bmjgh-2023-013245supp001.pdf]

Appendix S1 – Extracts from observation notes

Extract from Observation Note 23 [24-5-19]

Lemira kati village is on the highlands of Kilimanjaro with a foggy environment and a very cold weather. There is a road that is heading to Isuki and a feeder road into the village. The interview was done at a local political party office which is near the village offices, Church and a small business centre with a butcher around selling pork and several shops. There are a lot of banana plants around multiple block houses. Lots of motorcycles carry bananas down the road towards the village. The shops sell animal feeds and some there are some general retail shops. Opposite the CCM office is a SACCOSS [savings and credit organisation] group office together with coffee community office responsible for selling and buying coffee from around the villages, also a milling machine. Lemira is part of Masama Kati ward that comprises of several villages namely Mbosho, Mroma, Isuki, Nguni and Lemira Kati.

There is no health care facility in Lemira village. The services are found in the nearby villages inside the Masama Kati ward. Most people get health care from a pharmacy at Mroma village called [name of shop] where they get medical advice and some drugs. If they don't get relief then they go to Lemira Ngaya dispensary that is up near Isuki road. The dispensary only provides first aid and mostly refers patients to health centres like Masama and Nkwansira which is also on the Isuki road. The other referral is to the Hai district hospital or Machame hospital which is big hospitals in the District. Most people tend to copy medications that others used to clear similar symptoms and just find them from the pharmacy without actual medical consultation or advice. Also there are several local medications made from plants used to treat multiple conditions. Most people learn them from their grandparents.

Examples of dimensions of "thick description" as identified by Ponterotto, 2006: highlight key

Social context
Capturing thoughts
Assigning Intention and Motivation

Ponterotto, J. G. (2006). "Brief note on the origins, evolution, and meaning of the qualitative research concept thick description." The qualitative report 11(3): 538-549.
