# Supplementary material for: Rapid ethnographic appraisal of community concepts of and responses to joint pain in Kilimanjaro, Tanzania
Source: BMJ Glob Health. 2024 Jan 31;9(1):e013245. doi: 10.1136/bmjgh-2023-013245 (PMC10831465; doi:10.1136/bmjgh-2023-013245)
Supplement: Supplementary data [file bmjgh-2023-013245supp002.pdf]

Appendix S2 – Reflexivity Statement

1. How does this study address local research and policy priorities?

This research was part of a wider project exploring arthritis in the Kilimanjaro area, which was initiated by Prof Blandina Mmbaga, a researcher and clinician from the area, who sought out colleagues at the University of Glasgow to co-design a study to capable of generating policy-relevant data to guide clinical and decision-makers' response to the condition(s).

2. How were local researchers involved in study design?

The research design reported in this paper was conceived of through a four-way discussion between EFM, CB, EL and SW. EFM is a Tanzanian who lives and works in the region we studied and, as a co-investigator in the wider research grant, was keen to ensure that the project was well grounded in local understandings of joint pain. CB, an experienced social scientist who has supported and conducted researcher in Malawi, Kenya, South Africa, Zambia and Ghana, worked closely with EFM to construct a research design capable of doing justice to EFM's aim. EL and SW, also both experienced social scientists who have worked in a range of African countries, supported this process.

3. How has funding been used to support the local research team?

The wider project directly funded the research team in Tanzania, providing in the region of £1.2m over five years. As part of this funded activity, CB and EFM delivered training in qualitative data generation methods to eight members of KCRI's research staff. Following this, a broad-ranging interdisciplinary social science for health training package was delivered online (due to Covid-19 restrictions) to KCRI staff and made available as a digital resource hosted at KCRI for ongoing utilisation. Finally, the grant fully funded NY to study for an MSc in Global Health at University of Glasgow. Since this research, NY, PM and EFM have all enrolled in PhD programmes.

4. How are research staff who conducted data collection acknowledged?

All staff who collected data are included as authors.

5. Do all members of the research partnership have access to study data?

All members of the partnership have access to data.

6. How was data used to develop analytical skills within the partnership?

EFM, PM and CB analysed the data together through intensive, regular meetings throughout the research process, as described in the methods section of the paper. CB identified a qualitative analysis software package which was free to use to enable both EFM and PM to develop analytical skills that are transferable without access to expensive software subscriptions. Data analysis involved the sharing of these files and discussing interpretations together over video conferencing platforms, as a consequence of Covid-19 restrictions.

7. How have research partners collaborated in interpreting study data?

EFM, PM and CB led the analysis of the data through the regular meetings described above. This ensured Tanzanian analysts were the majority members of the primary analysis team. The wider authorship team were also brought into to interpretive discussions, ensuring that both Tanzanian (NY, BM) and UK-based (EL, SW, EM) researchers had the opportunity to interrogate the analysis we have presented.

8. How were research partners supported to develop writing skills?

CB worked closely to support EFM to co-author this paper. This was achieved through continuous discussion and mentorship, from the process of planning the paper, through to drafting each section. Two pre- doctoral early career researchers (PM and NY) on the authorship team were supported by senior academics to engage critically with and to contribute to the paper.

9. How will research products be shared to address local needs?

The findings from this research have been shared with members of the two communities through a dedicated community dissemination workshop held in Kilimanjaro during September, 2022. During the same week, findings were shared and deliberated with a diverse group of stakeholders including representatives from the Ministry of Health, leading Tanzanian clinicians, district commissioners and health officers and hospital managers. As part of this event, all attendees were provided with lay summaries of the findings from across the project, including the study presented in this paper.

10. How is the leadership, contribution and ownership of this work by LMIC researchers recognised within the authorship?

The joint first and senior authors are Tanzanians, reflecting leadership in identifying the need for the project (BM), the need for the specific study (EFM), and in the research process (EFM). EFM is the joint first author, recognising her equal contribution to the drafting of the manuscript (with CB) and PM, NY and BM are recognised as contributing to the revision and enhancement of the manuscript. EFM, PM and NY are recognised for their contributions as data generators, with EFM leading this process. Ownership of this research is recognised through EFM and CB being joint guarantors of the article.

11. **How have early career researchers across the partnership been included within the authorship team?**

Yes, three of the four Tanzanian researchers are early career researchers and all are listed as authors. None of the UK-based researchers are early career.

12. How has gender balance been addressed within the authorship?

One author is male (CB) and seven authors are female (EFM, PM, NY, EL, SW, EM and BM)

13. How has the project contributed to training of LMIC researchers?

See sections 3, 6, 7 and 8 – throughout this project, Tanzanian colleagues have been trained in qualitative data generation, analysis, software use and general social science for health perspectives.

14. How has the project contributed to improvements in local infrastructure?

This project has not directly contributed to improvements in local infrastructure.

15. What safeguarding procedures were used to protect local study participants and researchers?

Community entry was achieved through established, trusted, community partners who have long-term working relationships with KCRI. Standard operating, training and informed consent procedures were in place throughout the study to ensure that no participant or researcher was asked to do anything that they were uncomfortable with or was unsafe.
