# Supplementary material for: Rapid ethnographic appraisal of community concepts of and responses to joint pain in Kilimanjaro, Tanzania
Source: BMJ Glob Health. 2024 Jan 31;9(1):e013245. doi: 10.1136/bmjgh-2023-013245 (PMC10831465; doi:10.1136/bmjgh-2023-013245)
Supplement: Supplementary data [file bmjgh-2023-013245supp003.pdf]

S3 – Interview Sample

Location	Community Leaders		Religious Leaders		Traditional Healers		Drug Dispensers		Community Members 18-35		Community Members 35+	
	**M**	**F**	**M**	**F**	**M**	**F**	**M**	**F**	**M**	**F**	**M**	**F**
**Lemira Kati**	1	1	4	2	1	1	0	1	5	5	6	5
**Bomang'ombe**	1	1	3	2	1	1	2	1	5	5	5	7
**Totals**	**2**	**2**	**7**	**4**	**2**	**2**	**2**	**2**	**10**	**10**	**11**	**12**
