# Supplementary material for: Rapid ethnographic appraisal of community concepts of and responses to joint pain in Kilimanjaro, Tanzania
Source: BMJ Glob Health. 2024 Jan 31;9(1):e013245. doi: 10.1136/bmjgh-2023-013245 (PMC10831465; doi:10.1136/bmjgh-2023-013245)
Supplement: Supplementary data [file bmjgh-2023-013245supp004.pdf]

S3 - Typology of words and phrases collected during the REA

Word/Phrase	**Occurrence**	**Working Definition**	**Language**	**Usage notes based on perceptions captured in interview and focus group data**	**Likely or Unlikely Joint-Related?**
Ugonjwa wa baridi	*11*	*'Ugonjwa'* meaning diesease *'wa baridi'* meaning of cold	Swahili	The word is widely known and directly connected to joint pain. The word is used by and to refer to people who are suffering from joint pain, especially during cold season.	Likely
Ugonjwa wa Uzee	*8*	*'Ugonjwa'* meaning diesease '*Uzee'* meaning old age	Swahili	This word is common used by and about old people when speaking of joint pain.	Likely
Maumivu ya mguu	*7*	*'Maumivu'* meaning pain *'ya mguu'* meaning of legs	Swahili	Formal language commonly used when there is joint pain or an injury relating to the leg(s).	Likely
Nasikia maumivu ya viungo	*5*	*Nasikia'* meaning am feeling *'maumivu'* meaning pain *'ya viungo'* meaning of joints	Swahili	The word is widely known and used to speak of joint pain.	Likely
Magoti yanauma	*5*	*'Magoti'* meaning knee joints *'yanauma'* meaning pain	Swahili	This is the common word used to refer to joint pain in the knees.	Likely
Ugonjwa wa miguu	*4*	*Ugonjwa'* meaning disease *'wa miguu'* meaning legs	Swahili	The word is commonly used to express pain in the legs.	Likely
Ganzi	*4*	'Ganzi' loss of sensation and pain, attributed to a specific part of the body	Swahili	This is informal language, mostly used by younger people to describe loss of sensation and pain. The word can be used in relation to the joints.	Likely
Viungo vyote vinauma	*4*	*'Viungo'* meaning joints *'vyote'* meaning all *'vinauma'* meaning pain.	Swahili	Formal language, commonly used when a person is experiencing pain in all of the joints, often associated with systemic conditions.	Likely
Tambazi au chango	3	*'Tambazi'* meaning ?abscess au *'chango'* meaning ?abscess	Swahili	Traditional known word in the community, used to express cause of pain or disease from the body.	Unlikely
Baridi yabisi	*3*	*'Baridi'* meaning cold *'yabisi'* meaning dry/hard	Swahili	Often used to in relation to 'cold disease' (*ugonjwa wa baridi*) to describe joints which are inflexible.	Likely
Miguu inauma	*3*	*'Miguu inauma'*	Swahili	This is the common word used to express pains from the legs after walking a distance and get tired, the word is also connected to joint pain	Likely

Mgongo umekata	*3*	*Mgongo'* meaning backbone *'umekata'* meaning pain	Swahili	Informal language used to refer to back pain or backache.	Likely
Kiuno kinakata	*3*	*'Kiuno'* meaning waist *'kinakata'* meaning paining	Swahili	The world is common used by ladies during menstrual period, to describe pain experienced around the waist. The word is also commonly used by and about those in older age to speak of joint pain.	Likely
Mshipa ya miguu imeshika	*3*	*'Mshipa'* meaning muscles *'ya miguu'* of the legs '*imeshika'* meaning lock/tighten	Swahili	Informal language used when experiencing muscle cramps, also used by and about pregnant women when they experience muscle cramp.	Unlikely
Misuli inakamata	*3*	*'Misuli'* meaning muscles *'inakamata'* meaning lock/tighten	Swahili	Informal language used when experiencing muscle cramps, also used by and about pregnant women when they experience muscle cramp. Variation on *mshipa ya miguu imeshika.*	Unlikely
Maumivu ya mgongo	*2*	*Maumivu'* meaning pain *'ya mgongo'* meaning backbone.	Swahili	Commonly used by and to refer to men and women of all ages, specifically in relation to pain caused by doing heavy work, such as lifting heavy laggage. The word is used to speak of joint pain.	Likely
Najisikia kuchoka choka kama mlevi	*2*	*'Najisikia'* meaning feeling *'kuchoka choka'* meaning tiredness *'kama mlevi'* meaning like a drinker (alcohol user).	Swahili	This is the common word used to express general body pain, often connected to a disease such as Malaria.	Unlikely
Maumivu ndani ya mfupa	*2*	*'Maumivu'* meaning pains *'ndani ya mfupa'* meaning inside the bones.	Swahili	Informal language commonly used when a person is feeling pain relating to the bones. The word is used to speak of joint pain.	Likely
Magoti kuvimba	*2*	*'Magoti'* meaning knee *'kuvimba'* meaning swelling	Swahili	This is common and known word used to speak of joint pain/arthritis	Likely
Maumivu ya mgongo	*2*	*Maumivu'* meaning pain *'ya mgongo'* meaning of back=Backache.	Swahili	The word is common used when a person is having malaria,falling down, hard work,after delivery and old age.The condition is presented with the following symptoms such as legs	Likely
Mkono, miguu umekufa ganzi	*2*	*'Mkono'* meaning hands *'miguu'* meaning legs *'umekufa ganzi'* meaning loss of sensation/feeling	Swahili	The word is common used when the hands and/or leg joints lose senation, painfully.	Unlikely
Kuumwa na viungo	*1*	*'Kuumwa'* meaning pain *'na viungo'* meaning joints	Swahili	The word is commonly used and directly describes painful joints.	Likely

Maumivu ya mikono	*1*	*'Maumivu'* meaning pain *'ya mikono'* meaning of hand	Swahili	This is the word used to explain hand pain associated with hard work. Often gendered to refer to women.	Likely
Mifupa kutoboa	*1*	*Mifupa'* meaning bones *'kutoboa'* meaning aching	Swahili	The phrase is uncommon, but it is used to refer to joint pain.	Likely
Miguu inawaka moto	*1*	*Miguu'* meaning legs *'inawaka moto'* meaning feeling fire/pain	Swahili	The phrase is uncommon, but it is used, especially with older people, to speak of joint pains in the leg.	Likely
Kuumwa na pingili	*1*	*'Kuumwa'* meaning feel sick *'na pingili'* meaning back bones	Swahili	The phrase is uncommon. It is used to refer to back pain associated with malaria or meningitis.	Likely
Kuumwa mifupa ya nyonga	*1*	*'Kuumwa'* meaning feel sick *'mifupa'* meaning bones *'ya nyonga'* meaning bones in the groin/waist	Swahili	The phrase is commonly used by women during pregnancy.	Unlikely
Naumwa na uti wa mgongo	*1*	*Naumwa'* meaning am sick *'na uti wa mgongo'* meaning relating to spinal cord	Swahili	The phrase is uncommon. It is used to refer to back/joint pain when suffering from malaria.	Likely
Ulemavu	*1*	*'Ulemavu'* meaning disability	Swahili	This word is used to refer to a person who cannot work or cannot walk and is dependent on others for help.	Unlikely
Najisikia vibaya	*1*	*Najisikia'* meaning I am feeling *'vibaya'* meaning bad	Swahili	This phrase is commonly used to describe general feelings of illness and distress. This word is used to refer the bad feelings associated with joint pain.	Unlikely
Kiungo hakifanyi kazi	*1*	*Kiungo'* meaning joint; *'hakifanyi kazi'* meaning does not work	Swahili	This phrase is used is used to refer to joints which are not functioning, including painful joints. The phrase is not uncommon.	Likely
Paralize	*1*	*Paralize'* meaning paralysis	Swahili	The word is not common, but it is used when hands or legs are not moving properly. The word is also used in relation to joint pain.	Likely
Joint hakifanyi kazi	*1*	*Joint'* meaning joint *'hakifanyi kazi'* meaning does not work/function	Swahili	A common phrase used to describe a joint that is not functioning correctly, limiting movement. The phrase is also used in relation to joint pain.	Likely
Mguu umepigwa shoti	*1*	*'Mguu'* meaning legs 'umepigwa' meaning have got 'shoti' meaning spasms/cramps	Swahili	The phrase is commonly used to descibe a person who is experiencing muscle spasm/cramps that prevent walking. The phrase is also used in relation to joint pain.	Unlikely

Joint inashika	*1*	*'Joint'* meaning joints *'inashika'* meaning lock/tighting	Swahili	Informal language, commonly used by and to refer to people whose joints are stiff/not functioning. The phrase is also used in relation to joint pain.	Likely
Baridi imeingia kwenye viungo	*1*	*'Baridi'* meaning cold *'imeingia'* meaning getting in *'kwenye viungo'* meaning in the joints.	Swahili	Informal language commonly used during the cold season by and to refer to people who experience joint pain.	Likely
Viungo kukamata	*1*	*'Viungo'* meaning joints *'kukamata'* meaning locking /tighting/pain	Swahili	Informal language, commonly used to describe joints which are stiff or locked and is associated with joint pain.	Likely
Joint za mikono zinauma	*1*	*'Joint'* meaning joint *'za mikono'* meaning of the hands *'zinauma'* meaning are in pain.	Swahili	A phrase widely used to describe painful joints in the hand.	Likely
Naumwa ndani kwenye mifupa	*1*	*'Naumwa'* meaning I am sick *'ndani'* meaning inside *'kwenye mifupa'* meaning of the bones	Swahili	Informal language commonly used to speak of bone diseases and related joint pain	Likely
Mguu kushuka kwenye nyonga	*1*	*'Mguu'* meaning legs *'kushuka'* meaning tightening *'kwenye nyonga'* meaning on the waist/groin	Swahili	The phrase is commonly used by women during pregnancy. It is also connected to joint pain. See also *kuumwa mifupa ya nyonga*	Likely
Viungo vimechoka	*1*	'Viungo' meaning joints 'vimechoka' meaning tired	Swahili	This phrase is used to describe painful joints after significan exertion, such as lifting heavy items, climbing mountains or doing hard work.	Likely
Goti linavuta kwa ndani	*1*	*'Goti'* meaning knee *'linavuta'* meaning pulling *'kwa ndani'* meaning inside	Swahili	This phrase is commonly used to describe pain relating to the knee.	Likely
Kiungo kinavuta	*1*	*'Kiungo'* meaning joint *'Kinavuta'* meaning pulling/pain	Swahili	This phrase is used when a person expereinces pain from the joints while walking. The word is commonly used to refer to joint pain.	Likely
Miguu inapekecha	*1*	'Miguu' meaning legs 'inapekecha' meaning pain/ache	Swahili	This phrase is uncommon, but it is used to speak of joint pain relating to the legs	Likely
Nahisi vichomi	*1*	*'Nahisi'* meaning am feeling *'vichomi'* meaning burning pains	Swahili	The phrase is used to refer to stomach-ache, chest pain, and joint pain. It is uncommon, but it is used to speak of burning pain in the body.	Likely

Uchovu wa viungo	*1*	*'Uchovu'* meaning tiredness *'wa viungo'* meaning of joints	Swahili	The phrase is commonly used to speak of joint pain, especially when one has malaria or has worked hard.	Likely
Maradhi ya viungo	*1*	*'Maradhi'* meaning illness/diseases *'ya viungo'* meaning of joints	swahili	This phrase directly refers to pain and illness relatin gto the joints. The phrase is used by people who suffer from joint pain.	Likely
Ukosefu wa vitamin mwilini	*1*	'*Ukosefu wa*' meaning lack of '*vitamin mwilini'* meaning vitamins in the body	Swahili	The phrase is common used when a child or a young person develops problems with their bones or joints, attributing this to the absence of certain vitamins.	Likely
Nimeruka kitu kibaya	*1*	*'Nimeruka*' meaning I have been jumped by *'kitu'* meaning something *'kibaya'* meaning bad	Swahili	This phrase describes being attacked by witchcraft and is commonly used when a problem, such as an illness or symptom does not go away. It can be used in relation to joint pain.	Likely
Maumivu ya kifua	*1*	*Maumivu'* meaning pain *'ya kifua'* meaning of chest	Swahili	This phrase refers to chest pain caused by cigarette smoking, drinking alcohol, cold weather and allergies.	Unlikely
Maumivu ya miguu	*1*	*Maumivu'* meaning pain *'ya miguu'* of legs	Swahili	This phrase is used to describe weakness in the legs associated with not eating well and old age. Symptoms include failure to stand and walk. The word is widely used and directly connected to joint pain	Likely
Maumivu ya kiuno	*1*	*Maumivu'* meaning pain *'ya kiuno'* meaning waist	Swahili	The phrase is used to describe a uterus infection or sexually transmitted diseases. Symptoms include failure to stand. The phrase is also widely used and connected to joint pain.	Unlikely
Kichwa kinauma	*1*	*'Kichwa'* meaning head 'kinauma' meaning pain	Swahili	This phrase is common and is used when a person has a headache and can be associated with joint pain.	Unlikely
Ugonjwa wa presha	*1*	*Ugonjwa'* meaning disease *'wa presha'* meaning of blood pressure	Swahili	This is an informal phrase, widely used in the community when a person has high blood pressure. It is also associated with joint pain.	Unlikely
Ugonjwa wa sukari	*1*	*ugonjwa'* meaning disease *'wa sukari'* of diabetes	Swahili	This is an informal phrase, widely used in the community when a person has diabetes. It is also associated with joint pain.	Unlikely

Tambazi	*1*	*Tambazi'* meaning oedema	Swahili	A common word associated with joint pain. The disease is caused by excess fluid trapped in the body tissues, which can affect the joints.	Unlikely
Mikono kuwaka moto	*1*	*Mikono'* meaning hand *'kuwaka moto'* meaning burning sensation	Swahili	The phrase is common used to express nerve dysfunction in the hand due to nerve damage from an underlying condition. It is also associated with joint pain.	Likely
Goti linavimba	*1*	*'Goti'* meaning knee *'linavimba'* meaning swelling/inflamation	Swahili	This phrase is used to describe a swollen knee, with intermittent pain and a decreased range of motion.	Likely
Mguu kufa ganzi	*1*	*'Mguu'* meaning legs *'kufa ganzi'* meaning numbness	Swahili	This phrase is commonly used to refer to when a person experiences numbness after sitting in a position for a long period. The word is also used when a person is experiencing difficult walking.	Unlikely
Maumivu ya magoti	*1*	*Maumivu'* meaning pain *'ya magoti'* meaning knee joint	Swahili	This phrase is commonly used to express pain around the knee. The phrase is also connected to a lack of nutrients or old age.	Likely
Kifua kubana	*1*	*kifua'* meaning chest *'kubana'* meaning tightness	Swahili	This phrase is commonly used when a person is having problem with the chest such as asthma, TB or if smoking. The phrase describes difficultly in breathing.	Likely
Viungo kuwa na baridi	*1*	*Viungo'* meaning joints *'kuwa na baridi'* meaning have become cold	Swahili	This phrase is commonly used to describe pain in the joints, believed to be caused by the cooling of the joints. It is associated with joint pain that occurs during the cold season and in old age.	Likely
Miguu kuvimba	*1*	*Miguu'* meaning legs *'kuvimba'* meaning swelling or inflamation	Swahili	This phrase is commonly used when a person has swelling in their lower limbs due to disease such as kidney diseases. But it is also used to to describe swolen joints caused by gout.	Likely

Word/Phrase	**Occurrence**	**Working Definition**	**Language**	**Usage notes based on perceptions captured in interview and focus group data**	**Likely or Unlikely Joint-Related?**
Ndwari ya mbeho	3	*Ndwari'* meaning disease *'ya mbeho'* meaning of cold	Chagga	This phrase is widely known and is directly connected to joint pain. The word is used by and to refer to people who are suffering from joint pain, especially during cold season.	Likely
Nkewawio mungo	3	*Nkewawio'* meaning pain *'mungo'* meaning back bone	Chagga	This phrase is commonly used by older peole to express backpain due to old age. The word is also used to explain back pain caused by falling down, cold conditions and hard work.	Likely
Shibario mahende	3	*'Shibario'* meaning pain *'mahende'* meaning legs	Chagga	This phrase is commonly used to desribe pain and tiredness of the legs or discomfort in the legs. The phrase is associated with joint pain.	Likely
Nkeparwa mahende	*2*	'Nkeparwa' meaning pain 'marende' meaning legs	Chagga	This phrase is commonly used to desribe pain and tiredness of the legs or discomfort in the legs. The phrase is associated with joint pain. See also *shibario mahende*	Likely
Nkeparwa mungo	*2*	*'Nkeparwa'* meaning pain *'mungo'* meaning backbone	Chagga	This phrase is commonly used to desribe pain and discomfort in the back. The phrase is associated with joint pain.	Likely
Ugonjwa wa mbeho	*2*	*'Ugonjwa'* meaning disease *'wa mbeho'* meaning of cold	Chagga	This phrase is widely known and is directly connected to joint pain. The word is used by and to refer to people who are suffering from joint pain, especially during cold season. See also *n dwari ya mbeho*	Likely
Nshikewawio ndii	*2*	*'Nshikewawio'* meaning having pain *'ndii'* meaning knee joint	Chagga	This phrase is commonly used to describe pain or swelling on the knee. The phrase is associated with joint pain.	Likely

Madendee yakewawia	*2*	'Madendee' meaning legs 'yakewawia' meaning pains	Chagga	This phrase is commonly used to describe pain in the legs. The phrase is associated with joint pain.	Likely
Mungo keshiwaia	*2*	*Mungo'* meaning back bone *'keshiwaia'* meaning pain	Chagga	The phrase is commonly used to express back pain caused by falling down, hard work or old age.	Likely
Wakiwawio mahende	2	*Wakiwawio'* meanin pain *'mahende'* meaning legs	Chagga	The phrase is used to describe physical discomfort in part or all of the leg, or joint weakness, or burning sensation and or leg cramps. This phrase is not widely known, but it is directly connected to joint pain.	Likely
Nkebario maoko	2	*'Nkebario'* meaning pain *'maoko'* meaning hands	Chagga	This phrase is commonly used by and to refer to those (mostly women) who experience pain in the hands associated with over work. The word is directly connected to joint pain.	Likely
Nkeparwa maoko	*1*	*'Nkeparwa* ' meaning pain *'maoko'* meaning hands	Chagga	This phrase is commonly used to refer to those who experience pain in the hands. The word is directly connected to joint pain.	Likely
Maoko akewawa deny	*1*	*'Maoko'* meaning hands *'akewawa deny'* meaning having severe pain	Chagga	This phrase is commonly used to refer to those who experience severe pain in the hands after hard work. The word is directly connected to joint pain.	Likely
Mungo washiwawia denyi	*1*	Mungo' meaning back bone 'washiwawia denyi' meaning severe pain	Chagga	The phrase is commonly used to express severe back pain caused by bending down often, hard work or bearing a large number of children.	Likely
Malungo yakewawa	*1*	*'Malungo'* meaning joints *'Yakewawa'* meaning pain	Chagga	This phrase is commonly used to refer directly to pain in the joints.	Likely
Shikewawio marende	*1*	*Shikewawio'* meaning I am sick *'Marende'* meaning legs	Chagga	This phrase is used by Chagga people from the Massama area to describe painful legs, particularly related to fatigue.	Likely

Viungo vyako vikewawa	*1*	*Viungo vyako'* meaning my joints; *'vikewawa'* meaning pain	Chagga	This phrase is commonly used to refer directly to joint pain when a person is tired from work or when they have malaria.	Likely
Marende yakeshibaria deni	*1*	*’Marende'* meaning legs *'yakeshibaria deni’* meaning in a lot of pain	Chagga	This phrase is commonly used to refer to severe leg pains. The phrase is often specificly applied to problems with the leg bones.	Likely
Mawina	*1*	*mawina'* meaning joints pain	Chagga	The word is used to refer to recurring joint pain, especially during cold season. Also it is commonly used when a person experiences joint pain caused by previous accidents, again during the cold season. It is also commonly used by and to refer to older people who experience joint pain, especially during cold season.	Likely
Mbeyo	*1*	*'Mbeyo'* meaning pain in the shoulder	Chagga	The word refers to pain in the shoulder, particularly related to the cold. It is not widely used	Likely
Ndwali Nkuluwe	*1*	*Ndwali'* meaning any joint pain *'Nkuluwe'* meaning any joint pain	Chagga	These words are used interchangeably to express pain in the joints. They are often used specifically to describe the inflammation of joints or gout.	Likely
Mahende akewawa mdo	*1*	*Mahende'* meaning legs *'akewawa mdo'* meaning burning sensation	Chagga	This phrase is used to express burning sensations in the legs which is asscoiated with the cold season and is said to be common among older people.	Likely
Mungo kewawa	*1*	*Mungo'* meaning backbone *'kewawa'* meaning pain.	Chagga	The word is common known by Chagga people and used when a person is having malaria or falling down or hard work or after delivery and is mostly common expressed by old people with symptoms of legs weakness, fever condition.And	Likely
Ndori ambio	*1*	*Ndori* 'meaning disease *'ambio'* meaning cold	Chagga	This phrase is well known and used to describe joint pain that occurs during the cold season. The word is also used to express pain specifically in the hands, waist and shoulders.	Likely

Ndi kikeshiwaia	*1*	*Ndi'* meaning legs *'kikeshiwaia'* meaning pain	Chagga	This phrase is used to to express discomfort in part or all of the leg including weakness, burning sensations and cramps. The word is not widely known, but it is assoicated with joint pain.	Likely
Shikewawiode	*1*	*Shikewawiode'* meaning joint pain	Chagga	This phrase is used to refer to any pain in the joints, such as hands, knees, waist. It is not widely used. However, it is directly connected to joint pain.	Likely
Maoko akewawa	*1*	*Maoko'* meaning hands *'akewawa'* meaning pain	Chagga	This phrase is used to describe tenderness, swelling and stiffness relating to the hands and fingers. It is not widely known, but it is directly connected to joint pain. See also *Maoko akewawa deny*	Likely
Nukewawa	1	*Nukewawa'* meaning finger pain.	Chagga	This word is used to describe the loss of joint mobility in the fingers which impacts everyday activities. The word is not widely known but is directly connected to joint pain	Likely
Wakiwio mungo	1	*Wakiwio'* meaning pain *'mungo'* back bone	Chagga	This phrase is used to describe back pain caused by malaria, falling down, hard work or walking long distances. it is particularly applied to older people in whom it is associated with legs weakness and fevers. The word is not widely known but it is directly connected to joint pain.	Likely
Wakiwawio magoti	1	*Wakiwawio'* meaning pain *'magoti'* meaning knee joint	Chagga	This phrase is used to describe oain in or around the knee. The word is also used when a person is having difficults sitting or is experiencing sore joints. The phrase is not widely known but is directly connected to joint pain.	Likely

Mungo kebara	1	*Mungo'* meaning back bone *'kebara'* meaning pain	Chagga	This phrase is used used when a person expreriences backache after doing hard work. The word is directly related to joint pain	Likely
Nkewawio malungo	1	*Nkewawio'* meaning pain *'malungo'* meaning joints	Chagga	This phrase is commonly used to refer to any pain in the joints, such as hands, knees, waist. It is directly connected to joint pain.	Likely
Shite mbeyo	1	*Shite'* meaning feeling *'mbeyo'* meaning cold	Chagga	This phrase is commonly used during cold season to describe discomfort associated with the cold. It is widely used by and to refer to people who are suffering from joint pain. The phrase is directly associated with joint pain.	Likely
Maoko alemakaside	1	*'Maoko'* meaning hands *'alemakasidenyi'* meaning severe pain	Chagga	This phrase describes severe hand pains often connected to accidents or cold conditions. The word is most used by older people to speak of joint pain.	Likely

Word/Phrase	Occurrence	Working Definition	Language	Usage notes based on perceptions captured in interview and focus group data	Likely or Unlikely Joint-Related?
Faditilia	*1*	*'Faditilia'* meaning any joint pain.	Iraq	This word is commonly used to express any joint pain, but often in realtion to the hands, waist, legs or gout. The word is not widely known, but it is directly connected to joint pain.	Likely
Sagiktili Saga	*1*	*Sagiktili'* meaning pain *'Saga'* meaning head	Iraq	A commonly used phrase describing headaches. The word is also used to refer to joint pain in the head e.g. neck	Unlikely
Uratili	*1*	*Ura'* meaning stomach *'tili'* meaning pain	Iraq	This word is used to express pain from inside the stomach. The word is also used when a person has a muscle strain or constipation. The word is not widely used. However, it is not directly connected to joint pain.	Unlikely
Yahtili	*1*	*Yah'* meaning leg *'tili'* meaning pain	Iraq	This word is used to describe physical discomfort in the legs with symptoms of lower limb weakness, burning sensations and cramps. The word is not widely known, but is used to speak of joint pain.	Likely
Dabatili	*1*	*Dab' meaning hands 'atili'* meaning pain	Iraq	This word is used to describe pain in the hands, including stiffness and swelling. The word is widely known and is used to speak of joint pain.	Likely
Dandatili	*1*	*Danda'* meaning back bone *'tili'* meaning pain	Iraq	This word is used to describe backache caused by falling down, injury or hard work. The word is often used by older people, but is not widely known. It is directly connected to joint pain.	Likely
Yah tahamae	*1*	*'Yah'* meaning legs *'tahamae'* meaning pain	Iraq	This phrase is commonly used word to describe leg pain including weakness, burning sensation and cramps. The word is common and direct connected to arthritis. See also, *yahtili*	Likely

Word/Phrase	Occurrence	Working Definition	Language	Usage notes based on perceptions captured in interview and focus group data	Likely or Unlikely Joint-Related?
Ongorio	*1*	*Ongorio'* meaning backache.	Maasai	This word is commonly used to describe backache relating to malaria, falling down/injury, hard work, after giving birth and old age. The word is widely used and refers directly to joint pain.	Likely
Embolosi	*1*	*Embolosi'* meaning waist pain.	Maasai	This word is commonly used to describe pain in the waist caused by hard work, old age and cold conditions. The word is widely used and refers directly to joint pain.	Likely
Ngaeki	*1*	*Ngaeki'* meaning hand pain.	Maasai	This word is commonly used to describe pain in the hands. The word is widely used and refers directly to joint pain.	Likely
Ngaejeki	*1*	*Ngaejeki'* meaning leg pain.	Maasai	This word is commonly used to describe pain in the leg caused by weakness from not eating well/old age/hard work. It casuses problems with standing and walking.The word is used to refer to joint pain.	Likely
Elokonya	*1*	*'Elokonya'* meaning headache.	Maasai	This word is commonly used to describe pains in the head, which are sharp or dull. The word also is sometimes associated with joint pain.	Unlikely
Engohoke	*1*	*Engohoke'* meaning stomach ache.	Maasai	This word is commonly used to describe pain in the stomach, ranging from mild and temporary to severe. It is aso includes muscle strain and constipation. The word is not widely known and not directly connected to joint pain.	Unlikely

Word/Phrase	Occurrence	Working Definition	Language	Usage notes based on perceptions captured in interview and focus group data	Likely or Unlikely Joint-Related?
Naumiwa	*1*	*Naumiwa'* meaning I am sick	Sambaa	This word is used to speak of general sickness, including joint pain. It is connected to joint pain	Unlikely
Mavuha ya gwii	*1*	*Mavuha'* meaning bones *'ya gwii'* meaning mine	Sambaa	This phrase is commonly used to refer to bone pain or bone disease. The phrase can also be used after hard work or to describe a fracture or bone-related injury.	Likely
Mihundi	*1*	*Mihundi'* meaning legs pain.	Sambaa	This word is common used to express physical discomfort in the legs, characherized by weakness, burning sensations and cramps. The word is directly connected to joint pain	Likely
Miguu yaniuma	*1*	*Miguu'* meaning legs *'yaniuma'* meaning pain	Sambaa	This word is commonly used to describe leg pains relating to hard work, such as walking long distances or doing significant farm work. The word is also used when a person is sick and experiences pain in the legs. The word is directly connected to joint pain	Likely
Mwili woshe waniuma	*1*	*Mwili'* meaning body *'woshe'* meaning whole '*waniuma'* meaning pain.	Sambaa	This phrase is commonly used when a person is sick, has suffered injury from a fall, experiences pain from working hard or pain from exposure to cold conditions. The word also is used to speak of joint pain.	Unlikely

Word/Phrase	Occurrence	Working Definition	Language	Usage notes based on perceptions captured in interview and focus group data	Likely or Unlikely Joint-Related?
Natamwa	*1*	*'Natamwa'* meaning any joint pain.	Gogo	This word is commonly used to express any type of joint pain including in the hands, waist, back and legs. The word is directly connected to joint pain.	Likely
Migulu ifugamilo	*1*	*Migulu*' meaning knee joint *'ifugamilo'* meaning pain	Gogo	This phrase is used to express pain in or around the knee which. The word is also used when a person experiences pain from sitting on knees for a prolonged period or when the knee is swollen. The word is widely known, but is directly connected to joint pain.	Likely
Ukutamwa namgati nakuvimba	*1*	*Ukutamwa'* meaning pain *'namgati'* meaning knee*'nakuvimba'* meaning swelling	Gogo	This phrase is used to express pain in the knee joints. It is also used to describe a swollen knee, intermittent pain and decreased range of motion. The word is not widely known, but directly connected to joint pain.	Likely
Namtwe ukubwanhola	*1*	*Namtwe'* meaning *'ukubwanhola'* meaning hea	Gogo	This phrase is used when a person is suffering from a headache. The informant also express that, the word is also used to speak to joint pain.	Unlikely

Word/Phrase	Occurrence	Working Definition	Language	Usage notes based on perceptions captured in interview and focus group data	Likely or Unlikely Joint-Related?
Nolumwa mafindi	1	*'Nolumwa'* meaning am feeling sick *'mafindi'* meaning waist	Kwere	The phrase is commonly used to speak of waist pain. The pain can be associated with work or diseases. The word is sometimes connected to joint pain	Unlikely
Nolumwa mayot	1	Nolumwa' meaning feeling sick 'mayot' meaning knee joint	Kwere	The phrase is commonly used to describe pain or discomfort in the knee joins. The word is directly connected to joint pain	Likely
Vyoluma milono	1	'vyoluma' meaning pain 'milono' meaning wrist joint	Kwere	The phrase is commonly used to describe pain in the wrist. This can be attributed to fracture or disease. The word is directly connected to joint pain	Likely

Word/Phrase	Occurrence	Working Definition	Language	Usage notes based on perceptions captured in interview and focus group data	Likely or Unlikely Joint-Related?
Rimatizim	*6*	Rheumatism	English	This word is a localisation of the English word 'rheumatism' and is used to describe stiff and sore joints. The word is directly connected to joint pain and it is widely used.	Likely
Gauti	*5*	*'Gauti'* meaning disease of the lower limbs, especially the knee joints	English	This word is a localisation of the English word 'gout'. The word is widely know in the urban area, but less so in the rural area. 'Gauti' is associated with eating goat meat and with alcohol (beer). Other people associated the condition with dryness of the joints fluids especially the knee joints. The word is directly connected to joint pain	Likely
Malaria	*1*	*'Malaria'* meaning parasitic infection with plasmodium	English	This is the word commonly used to describe confirmed malaria. But is also used to speak of joint pain when malaria has not been confirmed.	Unlikely

Word/Phrase	Working Definition	Language	Usage notes based on perceptions captured in interview and focus group data	Likely or Unlikely Joint-Related?
Muhongo kukukusha	*Muhongo'* meaning back bone *'kukukusha'* meaning shaking	Pare	This phrase is commonly used to describe backache and is associated with shaking or spasms. The condition can be associated with condition such as malaria, or high fever. The word is also uesd to refer to joint pain.	Unlikely
Vivaju	*Vivaju'* meaning rib pain.	Pare	This word is commonly used to describe injury below the chest, including both dull and sharp pain. The word is not widely known but is indirectly connected to joint pain.	Unlikely

Word/Phrase	Working Definition	Language	Usage notes based on perceptions captured in interview and focus group data	Likely or Unlikely Joint-Related?
Muviri wakatire sana	*'Muviri'* meaning body *'wakatire sana'* meaning lots of pain	Rangi	This phrase is commonly used to describe body pain, such as when having fever due to malaria. It is also connected to joint pain.	Unlikely
Nukitera wakatire sana	*'Nukitera'* meaning joints *'wakatiri sana'* meaning lots of pain	Rangi	This phrase is commonly used to describeg severe joint pain. It is directly connected to joint pain.	Likely
